# Two-stage culture procedure using thidiazuron for efficient micropropagation of *Stevia rebaudiana*, an anti-diabetic medicinal herb

**DOI:** 10.1007/s13205-013-0172-y

**Published:** 2013-09-26

**Authors:** Pallavi Singh, Padmanabh Dwivedi

**Affiliations:** Laboratory of Plant Tissue Culture and Stress Physiology, Department of Plant Physiology, Institute of Agricultural Sciences, Banaras Hindu University, Varanasi, India

**Keywords:** Micropropagation, Murashige and Skoog medium, *Stevia rebaudiana*, Stevioside, Thidiazuron

## Abstract

*Stevia rebaudiana* Bertoni, member of Asteraceae family, has bio-active compounds stevioside and rebaudioside which taste about 300 times sweeter than sucrose. It regulates blood sugar, prevents hypertension and tooth decay as well as used in treatment of skin disorders having high medicinal values, and hence there is a need for generating the plant on large scale. We have developed an efficient micropropagation protocol on half strength Murashige and Skoog (MS) media, using two-stage culture procedures. Varying concentrations of cytokinins, i.e., benzylaminopurine, kinetin and thidiazuron (TDZ) were supplemented in the nutrient media to observe their effects on shoot development. All the cytokinins promoted shoot formation, however, best response was observed in the TDZ (0.5 mg/l). The shoots from selected induction medium were sub-cultured on the multiplication media. The media containing 0.01 mg/l TDZ produced maximum number of shoot (11.00 ± 0.40) with longer shoots (7.17 ± 0.16) and highest number of leaves (61.00 ± 1.29). Rooting response was best observed in one-fourth strength on MS media supplemented with indole-3-butyric acid (1.0 mg/l) and activated charcoal (50 mg/l) with (11.00 ± 0.40) number of roots. The plantlets thus obtained were hardened and transferred to the pots with soil and sand mixture, where the survival rate was 80 % after 2 months. Quantitative analysis of stevioside content in leaves of in vivo mother plant and in vitro plantlets was carried out by high performance liquid chromatography. A remarkable increase in stevioside content was noticed in the in vitro-raised plants as compared to in vivo grown plants. The protocol reported here might be useful in genetic improvement and high stevioside production.

## Introduction

*Stevia rebaudiana* Bertoni, a member of Asteraceae family, native to certain regions of South America-Brazil and Paraguay (Alhady 2011), is one of the important anti-diabetic medicinal herbs. It is indigenous to the Rio Monday Valley of the Amambay mountain region at altitudes between 200 and 500 m (Pande and Gupta 2013). The compounds in its leaves, stevioside and rebaudioside taste about 300 times sweeter than sucrose (Geuns 2003). It is used as sweetening agent and has enormous commercial importance. Its other medicinal uses include regulating blood sugar, preventing hypertension and tooth decay, and treatment of skin disorders (Singh and Rao 2005). *Stevia* also has healing effect on blemishes, wound cuts and scratches, besides being helpful in weight and blood pressure management. Conventional propagation in this plant is restricted due to the poor seed viability coupled with very low germination rate. Role of vegetative propagation method is also limited as specific habitat conditions are mandatory to grow the plants in addition to low acclimatization rate in soil. A suitable alternative method to prepare sufficient amount of plants within short time duration is the use of in vitro cultures. There are reports of in vitro clonal propagation of *Stevia* using nodal segments (Sivaram and Mukundan 2003; Mitra and Pal 2007; Singh et al. 2012). In vitro clonal propagation of *Stevia* has been carried out using nodal, inter-nodal segment (Uddin et al. 2006; Ahmed et al. 2007; Sairkar et al. 2009; Thiyagarajan and Venkatachalam 2012), leaf (Ali et al. 2010) and shoot-tip explants (Anbazhagan et al. 2010; Das et al. 2011). The present study was undertaken to evaluate the effectiveness of two-stage culture procedures as means of micropropagation of *S. rebaudiana* in half strength MS media, which has not been attempted before, using various cytokinins—benzylaminopurine (BAP), kinetin (Kn) and thidiazuron (TDZ). The study also compares the stevioside content in the in vivo and in vitro leaves, supporting the effectiveness of micropropagation protocol generated.

## Materials and methods

### Explant source

Healthy plants of *S. rebaudiana* were collected from local herbal nurseries and established in horticulture garden of BHU campus. Nodal explants (1.5–2.0 cm long and 0.2–0.4 cm thick) were washed thoroughly for 15 min under running tap water, treated with Tween 80 (2–3 drops in 100 ml) for 7 min followed by treatment with 0.002 % (w/v) bavistin for 2–3 min. These were surface sterilized with 0.1 % (w/v) HgCl_2_ for 2–3 min and washed 4–5 times with sterile double distilled water.

### Shoot induction media

Murashige and Skoog medium (1962) (half strength) was used supplemented with 3 % (w/v) sucrose, ascorbic acid (50 mg/l), gibberellic acid (1 mg/l), solidified with 0.8 % (w/v) agar; pH was adjusted to 5.8 prior to autoclaving at 121 °C for 20 min. The nodal explants were inoculated with different concentrations of TDZ (0.01, 0.03, 0.05, 0.1, 0.2, 0.5 mg/l), BAP (0.2, 0.5, 1.0 mg/l) and Kn (0.2, 0.5, 1.0 mg/l) to observe their effect on shoot development and multiplication. The cultures were kept under cool, fluorescent light (16 h photoperiod) at 25 ± 2 °C in the culture room. Data were noted after 3 weeks of inoculation.

### Multiplication media

Shoots (3-week-old) obtained from the shoot induction media containing 0.2 mg/l BAP (B), 0.2 mg/l Kn (E), 0.2 mg/l each of BAP and Kn (H), 0.5 mg/l TDZ (P), as shown in Table 1, were sub-cultured on multiplication media. The multiplication medium consisted of half MS supplemented with TDZ (0.01 mg/l); half MS devoid of any growth regulator and original shoot induction medium (B, E, H and P), as shown in Table 2; cultures without growth regulators served as control. Total number of shoots, length of shoots and number of leaves were observed after 4 weeks of culture. 

### In vitro root induction

The elongated shoots with length more than 5.0 cm were excised from the culture flasks and transferred to the rooting media amended with 0.2, 0.5 and 1.0 mg/l IBA under aseptic condition. The growth regulator was added separately to half strength and one-fourth strength MS media containing 3 % sucrose to determine the effect of MS salt concentration on root induction (Table 3). The media was additionally supplemented with activated charcoal (50 mg/l), ascorbic acid (50 mg/l), polyvinylpolypyrrolidone (100 mg/l) and gibberellic acid (0.5 mg/l). Total number of roots per shoot as well as length of the roots was measured after 4 weeks of culture.

### Hardening

Rooted plants were carefully removed from the culture flasks, washed with sterile water to remove agar media, placed in the plastic cups filled with sterilized perlite. The plants were covered with polythene bags to maintain high humidity. These plants were maintained in the culture room for 3 weeks with the following atmospheric conditions: temperature, 25 ± 2 °C; light, 16 h photoperiod. Afterwards these were transferred to pots containing sterilized garden soil and sand (1:1).

### Observation recorded and statistical analysis

Observations were recorded and different parameters (number of shoots, length of shoots and number of leaves) were examined using 8–10 replicates. Data were subjected to Duncan’s multiple range test (Duncan 1955).

### Stevioside analysis

Biochemical analysis of the in vivo and in vitro-raised plants was performed using more sensitive and rapid method of HPLC (Shimadzu, Japan) analysis to confirm the presence of stevioside in leaves. Stevioside standard was obtained from Sigma Ltd., USA (95 % purity).

Conditions for HPLC study: column used C-18, mobile phase used methanol:water (80:20) with the flow rate of 1.5 ml/min (injection 10 μl). Peak detection was made at 210 nm at the room temp of 25 °C.

Standard preparation: 1 mg of stevioside sample was dissolved in 10 ml of methanol. Then 10 μl was applied to HPLC chromatogram.

Sample preparation from *Stevia* leaves: *Stevia* leaves were dried at 50 °C for 24 h in dark and pulverized to uniform size. This material (2 g) was extracted in boiling water (2 × 50 ml) and further boiled for 30 s. Cooled extract was first filtered through the filter paper and then microfiltered (0.45 μm) before it was ready for analysis.

Calculation of percentage of stevioside (*X*) in the sample was done as per the formula*:

%X=[WS/W]×[fX×AX/AS]×100

where, WS is the amount (mg) of stevioside in the standard solution *W* is the amount (mg) of sample in the sample solution AS is the peak area for stevioside from the standard solution A*X* is the peak area of *X* for the sample solution f*X* is the ratio of the formula weight of *X* to the formula weight of stevioside: 1.00 (stevioside).

Prepared at the 68th Joint FAO/WHO Expert Committee on Food Additives JECFA (2007).

## Results and discussion

### Shoot induction

The nodal explants were inoculated in half strength MS media with various concentration of BAP (0.2, 0.5, 1.0 mg/l). Almost at every concentration, bud break was seen during 3–7 days after inoculation. The parameters recorded (number of shoots, length of shoot and number of leaves) have shown differences at various concentrations in the induction media. 0.2 mg/l BAP showed better growth in terms of number of shoots (2.25 ± 0.25), shoot length (1.20 ± 0.27 cm), number of leaves (15.75 ± 1.84). Multiple shoot observed from 1.0 to 2.0 mg/l BAP (MS media) and maximum response as well as healthy shoot was noticed at 1 mg/l BAP (Ranganathan 2012). When kinetin was used, 0.2 mg/l showed better response in terms of all the parameters; number of shoots (2.00 ± 0.00), shoot length (1.95 ± 0.35 cm), though media with 0.5 mg/l of kinetin has shown higher number of leaves (20.00 ± 0.81). BAP and Kinetin at different concentrations (0.2, 0.5, 1.0 mg/l of each) when applied together, produced the best response at lower concentration (0.2 mg/l), in terms of number of shoots (2.00 ± 0.00), shoot length (3.10 ± 0.96 cm) and number of leaves (11.50 ± 0.50). Mehta et al. (2012) reported that best shooting response was observed on MS media containing 0.5 mg/l BAP + 2.0 mg/l Kn (average number of shoots 3.42 ± 0.39) and 0.5 mg/l BAP + 0.5 mg/l Kn (average shoot length 7.54 ± 0.31 cm). BAP (0.2 mg/l alone) and BAP/Kn (0.5 mg/l each) showed same result in terms of number of shoots, but considering all the parameters together, BAP (0.2 mg/l) produced the best response in shoot induction media (Table 1).

**Table 1:** Effect of various cytokinins on in vitro shoot induction in *Stevia rebaudiana*

	Plant growth regulator (mg/l)			Number of shoots	Shoot length (cm)	Number of leaves
	BAP	Kinetin	TDZ			
A	0.0	0.0	0.0	1.50 ± 0.28^a^	1.75 ± 0.14^bcd^	14.50 ± 1.50^bc^
B	0.2	–	–	2.25 ± 0.25^a^	1.20 ± 0.27^abc^	15.75 ± 1.84^bc^
C	0.5	–	–	1.75 ± 0.25^a^	0.80 ± 0.08^ab^	15.25 ± 1.79^bc^
D	1.0	–	–	2.00 ± 0.00^a^	0.45 ± 0.09^a^	12.00 ± 1.63^ab^
E	–	0.2	–	2.00 ± 0.00^a^	1.95 ± 0.35^cde^	12.25 ± 0.25^ab^
F	–	0.5	–	1.50 ± 0.28^a^	1.00 ± 0.08^ab^	20.00 ± 0.81^de^
G	–	1.0	–	1.75 ± 0.25^a^	1.15 ± 0.05^abc^	17.50 ± 0.50^cd^
H	0.2	0.2	–	2.00 ± 0.00^a^	3.10 ± 0.96^f^	11.50 ± 0.50^ab^
I	0.5	0.5	–	2.25 ± 0.25^a^	0.82 ± 0.19^ab^	9.50 ± 0.95^a^
J	1.0	1.0	–	1.75 ± 0.25^a^	0.95 ± 0.12^ab^	8.00 ± 1.63^a^
K	–	–	0.01	2.00 ± 0.00^a^	2.82 ± 0.11^ef^	22.75 ± 0.94^ef^
L	–	–	0.03	2.00 ± 0.00^a^	2.60 ± 0.21^def^	23.50 ± 1.25^ef^
M	–	–	0.05	2.00 ± 0.00^a^	2.77 ± 0.13^ef^	24.25 ± 0.47^f^
N	–	–	0.1	2.00 ± 0.00^a^	2.65 ± 0.12^def^	24.75 ± 0.94^fg^
O	–	–	0.2	2.00 ± 0.00^a^	2.30 ± 0.13^def^	28.50 ± 1.25^g^
P	–	–	0.5	3.00 ± 0.57^b^	2.20 ± 0.11^def^	33.00 ± 2.88^h^

Parameters have been recorded after 3 weeks of culture. Data are in the form of mean ± SEM, and means followed by the same letter within the columns are not significantly different (*P* = 0.05) using Duncan’s multiple range test

**Fig. 1:**
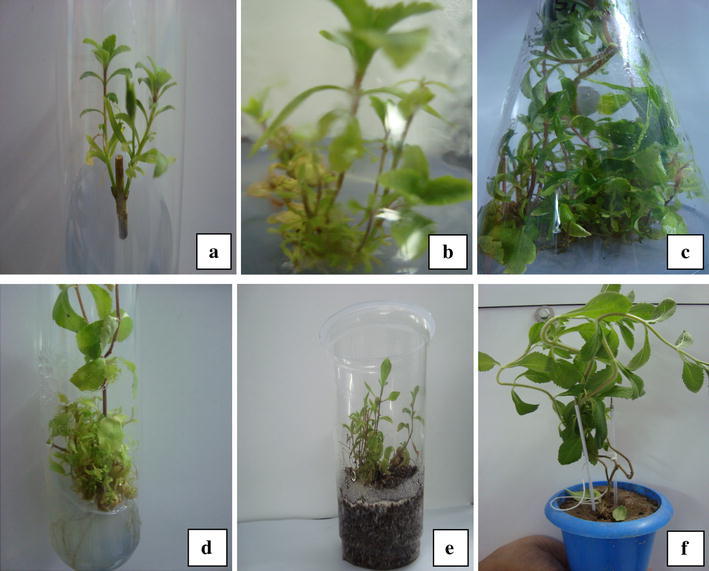
In vitro shoot multiplication and rooting of *Stevia rebaudiana.***a** Shoot formation in ½ MS supplemented with 0.01 mg/l TDZ. **b** Cultures in induction media TDZ (0.5 mg/l) transferred to ½ MS multiplication media without hormone. **c** Cultures in induction media TDZ (0.5 mg/l) transferred to ½ MS multiplication media supplemented with TDZ (0.01 mg/l). **d** In vitro rooting in ¼ MS supplemented with IBA. **e** Hardened plants in perlite. **f** In vitro-raised *Stevia rebaudiana* in garden soil and sand mixture

Nodal explants inoculated on half strength MS media having concentration of TDZ (0.01, 0.03, 0.05, 0.1, 0.2, 0.5 mg/l) showed shoot development at every concentration (Fig. 1a); 0.5 mg/l was found to be the most effective, i.e., number of shoots (3.00 ± 0.57), shoot length (2.20 ± 0.11 cm) and number of leaves (33.00 ± 2.88) (Table 1). It was noted that at lower concentration of TDZ, number of shoots was less and shoot length high, and on increasing the concentration, multiple shooting increases, but with decreased shoot length. Mithila et al. (2003) reported that low concentration of TDZ induced shoot organogenesis of African violet explants, whereas at higher doses (5–10 μM) somatic embryos were formed. Among other agents with cytokinin activity, comparatively low amount of TDZ promotes shoot multiplication in several plants (Guo et al. 2011). It was also reported that TDZ induced better response than BA (6-benzyl adenine) in shoot regeneration in peanut (Victor et al. 1999; Gairi and Rashid, 2004). Role of TDZ in shoot induction and multiple shooting has been reported in other plants as well; the highest rate of shoot regeneration from *Echinacea purpurea* leaf explants cultured on medium with TDZ at 2.5 μM or higher was reported (Jones et al. 2007). TDZ, a phenyl-urea type plant growth regulator, was earlier used as a cotton defoliant (Arndt et al. 1976). Later, it was believed to exhibit strong cytokinin-like activity almost similar to that of *N*6-substituted adenine derivatives (Mok et al. 1982; Gyulai et al. 1995). In the present study, TDZ appears to mimic cytokinin-like activity causing the release of lateral buds (Wang et al. 1986) and showed better response in terms of shoot regeneration efficiency, compared to other cytokinins, similar to other findings where TDZ produced shoots comparable to or greater than that of other cytokinins (Fiola et al. 1990; Malik and Saxena 1992).

At initial stage, best response of shoot induction, considering all the parameters together was observed at BAP (0.2 mg/l), Kn (0.2 mg/l), BAP/Kn (0.2 mg/l each) and TDZ (0.5 mg/l) (Table 1). These best concentrations, respectively, denoted as B, E, H and P were selected and transferred to different shoot multiplication media (Table 2).

**Table 2:** Effect of cytokinins on shoot multiplication of *Stevia rebaudiana*

Induction medium	Multiplication medium	Number of shoots	Shoot length (cm)	Number of leaves
BAP (0.2 mg/l)	B	5.75 ± 0.25^bc^	4.32 ± 0.48^a^	46.25 ± 0.62^cd^
	½ MS	3.25 ± 0.62^a^	4.20 ± 0.16^a^	37.50 ± 1.70^b^
	½ MS + TDZ (0.01)	9.25 ± 0.25^d^	6.67 ± 0.26^d^	50.50 ± 0.95^ef^
Kn (0.2 mg/l)	E	4.75 ± 0.25^b^	5.05 ± 0.09^b^	44.75 ± 0.48^c^
	½ MS	2.50 ± 0.28^a^	4.82 ± 0.16^ab^	30.50 ± 0.95^a^
	½ MS + TDZ (0.01)	8.50 ± 0.28^d^	6.95 ± 0.95^d^	50.00 ± 0.81^ef^
BAP and Kn (0.2 mg/l each)	H	5.25 ± 0.25^bc^	5.20 ± 0.14^b^	40.75 ± 0.75^b^
	½ MS	3.00 ± 0.40^a^	5.90 ± 0.12^c^	30.50 ± 1.70^a^
	½ MS + TDZ (0.01)	8.75 ± 0.25^d^	8.05 ± 0.22^e^	49.00 ± 1.29^de^
TDZ (0.5 mg/l)	P	6.00 ± 0.40^c^	4.85 ± 0.12^ab^	52.50 ± 1.25^f^
	½ MS	4.75 ± 0.25^b^	5.02 ± 0.16^b^	40.00 ± 0.81^b^
	½ MS + TDZ (0.01)	11.00 ± 0.40^e^	7.17 ± 0.16^d^	61.00 ± 1.29^g^

Parameters have been recorded after 4 weeks of transfer in multiplication media. Data are in the form of mean ± SEM, and means followed by the same letter within the columns are not significantly different (*P* = 0.05) using Duncan’s multiple range test

### Shoot multiplication

Shoots cultured in the same induction medium B, E, H, P and half MS medium devoid of growth regulator (Fig. 1b) produced minimal response of shoot multiplication; however, those sub-cultured in the half MS multiplication medium containing TDZ produced significantly higher number of shoots (Fig. 1c), the best response being observed in TDZ (0.01 mg/l). Shoots obtained from induction medium P containing TDZ (0.5 mg/l) produced highest number of shoots (11.00 ± 0.40) in half MS multiplication media containing 0.01 mg/l TDZ (Table 2). However, shoot obtained from the induction medium B containing BAP (0.2 mg/l), E containing Kn (0.2 mg/l) and H containing BAP/Kn (0.2 mg/l each), produced (9.25 ± 0.25), (8.50 ± 0.28), (8.75 ± 0.25) shoots, respectively, when transferred separately in MS half containing 0.01 mg/l TDZ. The number of shoots obtained is lesser when compared with those obtained from medium P containing 0.5 mg/l TDZ (Table 2).

### Shoot elongation

Shoot length increased in all the media supplemented with TDZ (Fig. 1c). Shoot obtained from induction medium H (BAP/Kn 0.2 mg/l each) showed highest shoot length (8.05 ± 0.22) when transferred in half MS multiplication medium supplemented with 0.01 mg/l TDZ (Table 2). It is to be noticed that there is a minute difference in shoot length of cultures obtained from induction medium H (BAP/Kn 0.2 mg/l each) and P (TDZ 0.5 mg/l), when they were transferred separately in half MS medium supplemented with 0.01 mg/l TDZ. The shoots sub-cultured in the same induction medium (B, E, H, P) and half MS medium devoid of growth regulator (Fig. 1b) did not significantly increase the length of the shoots (Table 2). Also, it is observed that shoot cultured in the medium containing TDZ produced significantly more number of leaves (Fig. 1c). Highest number of leaves (61.00 ± 1.29) was observed in the multiplication media with ½ MS + TDZ (0.01 mg/l), the shoots when cultured in the same induction medium (B, E, H, P) and half MS medium devoid of growth hormone showed comparatively lesser number of leaves, i.e., in the range of 30–52 leaves (Table 2).

### Root induction and hardening

Elongated shoots were separated from the shoot multiplication media and transferred to the rooting media. Root induction was observed in all cultures supplemented with different concentrations of IBA (Fig. 1d). IBA added to one-fourth MS medium produced a better rooting response as compared to half MS (Table 3). Highest number of roots (11.00) with longer roots (4.62 cm) was obtained on one-fourth MS medium supplemented with 1.0 mg/l IBA (Table 3). Use of activated charcoal in the rooting media facilitated rooting, as also reported in other studies (Komalivalli and Rao 2000; Priyadarshini et al. 2007). Rooted plants were hardened in perlite (Fig. 1e) where all of them were healthy and viable for 3 weeks in the culture room. The survival rate of the transferred rooted plants into the pots containing garden soil and sand (1:1) was 80 %, after 2 months (Fig. 1f). Two-stage culture procedure seems to be a versatile protocol for efficient induction and multiple shoot formation, in *Stevia* similar to that noticed in *Cassia angustifolia* (Iram and Anis 2007) and *Pterocarpus marsupium* (Husain et al. 2007). A two-stage culture procedure has been developed for highly efficient shoot regeneration from leaf and internode explants of *Bacopa monnieri* (Ceasar et al. 2010).

**Table 3:** Effect of MS salt concentration and IBA on in vitro root induction

Rooting media	IBA (mg/l)	Number of roots	Root length (cm)
½ MS media	0.2	1.75 ± 0.25^a^	1.85 ± 0.09^a^
	0.5	3.25 ± 0.25^b^	1.87 ± 0.11^a^
	1.0	6.00 ± 0.40^c^	2.20 ± 0.18^a^
¼ MS media	0.2	2.75 ± 0.25^ab^	2.12 ± 0.11^a^
	0.5	6.25 ± 0.85^c^	2.80 ± 0.18^b^
	1.0	11.00 ± 0.40^d^	4.62 ± 0.19^c^

Parameters have been recorded after 4 weeks of transfer in rooting media. Data are in the form of mean ± SEM, and means followed by the same letter within the columns are not significantly different (*P* = 0.05) using Duncan’s multiple range test

### Stevioside content

The presence of the active principles was confirmed in both the in vivo- and in vitro-derived leaves of *Stevia*. The identification and quantification of stevioside content in the samples were done by comparing the retention time and peak area of sample with that of the standard. In samples of in vitro plants, HPLC analysis revealed that stevioside content was higher than those of in vivo plants. Initial study showed that stevioside production was tissue and age dependent (data not shown). The percentage stevioside content of the samples, in the in vivo and in vitro leaves of *Stevia* was found to be 7.017 ± 0.058 and 9.236 ± 0.046, respectively (Table 4); the in vitro-raised plants had higher stevioside content.

**Table 4:** Stevioside content in in vivo and in vitro leaf samples

S. no.	Samples	Medium	Retention time (mm:ss)	Peak area (mAs)	Stevioside content (%)
1	Standard	–	2:22	596.1	–
2	In vivo plants	–	2:21	8,421.6	7.017 ± 0.058
3	In vitro plants (3-week-old)	Half strength MS	2:24	11,040.7	9.236 ± 0.046
4	In vitro-raised plants (8-week-old)	Half strength MS	2:24	11,040.7	9.236 ± 0.046

Two replicates of each sample were used for HPLC analysis (mean value calculated)

In conclusion, the present study demonstrates an efficient micropropagation protocol of *S. rebaudiana* using two-stage culture procedures. Furthermore, the micropropagation protocol generated did not affect the content of stevioside in *Stevia* leaves as shown by its higher content in the in vitro-raised leaves. The protocol might be useful in germplasm conservation and stevioside production.

## Abbreviations

MS: Murashige and Skoog
BAP: Benzylaminopurine
Kn: Kinetin
TDZ: Thidiazuron
IBA: Indole-3-butyricacid
HPLC: High performance liquid chromatography

## References

[CR1] Ahmed MB, Salahin M, Karim R, Razvy MA, Hannan MM, Sultana R, Hossain M, Islam R (2007). An efficient method for in vitro clonal propagation of newly introduced sweetener from plant *Stevia rebaudiana* Bertoni in Bangladesh. Am-Eurasian J Sci Res.

[CR2] Alhady MRAA (2011). Micropropagation of *Stevia rebaudiana* Bertoni—a new sweetening crop in Egypt. Glob J Biotechnol Biochem.

[CR3] Ali A, Gull I, Naz S, Afghan S (2010). Biochemical investigation during different stages of in vitro propagation of *Stevia rebaudiana*. Pak J Bot.

[CR4] Anbazhagan M, Kalpana M, Rajendran R, Natarajan V, Dhanavel D (2010). In vitro production of *Stevia rebaudiana* Bertoni. Emir J Food Agric.

[CR5] Arndt F, Rusch R, Stillfried HV (1976). SN 49537, a new cotton defoliant. Plant Physiol.

[CR6] Ceasar SA, Maxwell SL, Prasad KB, Karthigan M, Ignacimuthu S (2010). Highly efficient shoot regeneration of *Bacopa monnieri* (L.) using a two-stage culture procedure and assessment of genetic integrity of micropropagated plants by RAPD. Acta Physiol Plant.

[CR7] Das A, Gantait S, Mandal N (2011). Micropropagation of an elite medicinal plant: *Stevia rebaudiana* Bert. Int J Agric Res.

[CR8] Duncan DB (1955). Multiple range and multiple *F*-tests. Biometrics.

[CR9] Fiola JA, Hassan MA, Swartz HJ, Bors RH, McNicols R (1990). Effect of thidiazuron, light fluence rates and kanamycin on in vitro shoot organogenesis from excised *Rubus* cotyledons and leaves. Plant Cell Tissue Org Cult.

[CR10] Gairi A, Rashid A (2004). Direct differentiation of somatic embryos on different regions of intact seedlings of *Azadirachta* in response to thidiazuron. J Plant Physiol.

[CR11] Geuns JMC (2003). Molecules of interest: Stevioside. Phytochemistry.

[CR12] Guo B, Abbasi BH, Zeb A, Xu LL, Wei YH (2011). Thidiazuron: a multi-dimensional plant growth regulator. Afr J Biotechnol.

[CR13] Gyulai G, Jekkel Z, Kiss J, Heszky LE (1995). A selective auxin and cytokinin bioassay based on root and shoot formation in vitro. J Plant Physiol.

[CR14] Husain MK, Avis M, Shahzad A (2007). In vitro propagation of Indian kino (*Pterocarpus marsupium* Roxb.) using thidiazuron. In Vitro Cell Dev Biol Plant.

[CR15] Iram S, Anis M (2007). In vitro shoot multiplication and plantlet regeneration from nodal explants of *Cassia angustifolia* Vahl. a medicinal plant. Acta Phys Plant.

[CR16] JECFA (2007) Prepared at the 68th JECFA (2007) and published in FAO JECFA Monographs 4 (2007), superseding tentative specifications prepared at the 63rd JECFA (2004), in the combined compendium of food additive specifications, FAO JECFA Monographs 1 (2005)

[CR17] Jones MP, Yi Z, Murch SJ, Saxena PK (2007). Thidiazuron-induced regeneration of *Echinacea purpurea* L.: micropogation in solid and liquid culture systems. Plant Cell Rep.

[CR18] Komalivalli N, Rao MV (2000). In vitro micropropagation of *G. sylvestre*—a multipurpose medicinal plant. Plant Cell Tissue Org Cult.

[CR19] Malik KA, Saxena PK (1992). Regeneration of *Phaseolus vulgaris* L. High-frequency induction of direct shoot formation in intact seedlings by *N*6-benzylaminopurine and thidiazuron. Planta.

[CR20] Mehta J, Sain M, Sharma DR, Gehlot P, Sharma P, Dhaker JK (2012). Micropropagation of an anti diabetic plant—*Stevia rebaudiana* Bertoni (natural sweetener) in Hadoti region of south-east Rajasthan, India. ISCA J Biol Sci.

[CR21] Mithila J, Hall JC, Victor JMR, Saxena PK (2003). Thidiazuron induces shoot organogenesis at low concentrations and somatic embryogenesis at high concentrations on leaf and petiole explants of African violet (*Saintpaulia ionantha* Wendl.). Plant Cell Rep.

[CR22] Mitra A, Pal A (2007). In vitro regeneration of *Stevia rebaudiana* Bert. from the nodal explants. J Plant Biochem Biotechnol.

[CR23] Mok MC, Mok DWS, Armstrong DJ, Shudo K, Isogai Y, Okamoto T (1982). Cytokinin activity of *N*-phenyl-*N*-1,2,3-thiadiazol-5-ylurea (thidiazuron). Phytochemistry.

[CR24] Murashige T, Skoog F (1962). A revised medium for rapid growth and bioassays with tobacco tissue cultures. Physiol Plant.

[CR25] Pande SS, Gupta P (2013). Plant tissue culture of *Stevia rebaudiana* (Bertoni): a review. J Pharmacogn Phytother.

[CR26] Priyadarshini GR, Kumar A, Janifer X, Kukreja AK, Mathur AK, Banerjee S, Mathur A, Sharma A, Khanuja SPS (2007). Micropropagation studies in *Stevia rebaudiana* Bertoni. Proceedings of national symposium on plant biotechnology: new frontiers.

[CR27] Ranganathan J (2012). Studies on micropropagation of *Stevia rebaudiana*. Int J Pharmacol Biol Arch.

[CR28] Sairkar P, Chandravanshi MK, Shukla NK, Mehrotra MN (2009). Mass propagation of an economically important medicinal plant *Stevia rebaudiana* using in vitro propagation technique. J Med Plants Res.

[CR29] Singh SD, Rao GP (2005). *Stevia*: the herbal sugar of 21st century. Sugar Technol.

[CR30] Singh P, Dwivedi P, Atri N (2012). In vitro shoot regeneration of *Stevia rebaudiana* through callus and nodal segments. Int J Agric Environ Biotechnol.

[CR31] Sivaram L, Mukundan U (2003). In vitro culture studies on *Stevia rebaudiana*. In Vitro Cell Dev Biol Plant.

[CR32] Thiyagarajan M, Venkatachalam P (2012). Large scale in vitro propagation of *Stevia rebaudiana* Bert. for commercial application: pharmaceutically important and antidiabetic medicinal herb. Ind Crops Prod.

[CR33] Uddin MS, Chowdhury MS, Khan MM, Uddin MB, Ahmed R, Betan MA (2006). In vitro propagation of *Stevia rebaudiana* Bert in Bangladesh. Afr J Biotechnol.

[CR34] Victor JMR, Murthy BNS, Murch SJ, KrishnaRaj S, Saxena PK (1999). Role of endogenous purine metabolism in thidiazuron-induced somatic embryogenesis of peanut (*Arachis hypogaea*). Plant Growth Regul.

[CR35] Wang SY, Steffens GL, Faust M (1986). Breaking bud dormancy in apple with a plant bioregulator, thidiazuron. Phytochemistry.

